# The good, the bad, and the in-between: an exploratory study of how different groups may be represented in the brain

**DOI:** 10.3389/fnbeh.2026.1773300

**Published:** 2026-05-29

**Authors:** Jeroen Delplanque, Frank Van Overwalle

**Affiliations:** Psychology and Educational Sciences, Vrije Universiteit Brussel, Brussels, Belgium

**Keywords:** fMRI, repetition suppression, social cognition, social groups, social neuroscience, stereotype content model

## Abstract

**Introduction:**

Social categorization is a fundamental aspect of social perception, enabling individuals to process vast amounts of information efficiently. This exploratory fMRI study investigated whether different types of social groups are represented in the brain as stable memory representations.

**Methods:**

We used repetition suppression as a proxy-measurement for such stable representation, examining four groups categorized by their warmth and competence into the quadrants of the Stereotype Content Model (SCM). Participants judged the typicality of behavioral sentences associated with these groups, presented in pairs that either repeated the same group, or not.

**Results:**

A repetition suppression pattern was observed in the posterior temporal sulcus (pSTS) for groups with mixed warmth and competence; specifically the rich (low warmth, high competence) and the poor (high warmth, low competence). By contrast, no suppression was detected for non-mixed groups (military people and welfare recipients).

**Discussion:**

We theorize that mixed groups elicit greater ambiguity, which may prompt the brain to form more robust representations to quickly assess their functional relevance. These findings suggest that stable group representations are not uniformly applied across all group types and may reflect social complexity and motivational salience. The results highlight the pSTS as a key node in group representation and invite further research into its functional role.

## Highlights

- fMRI repetition suppression isolates neural areas which encode information in stable representations.
- This technique identified representations in the middle temporal gyrus for groups of mixed warmth and competence (i.e., the Rich and the Poor).

## Introduction

A fundamental feature of our social perception is the categorization of other persons into groups (Brewer, 1988; Tajfel et al., 1971; Turner et al., 1987). By organizing our social surroundings, including other humans, we are better able to grasp an otherwise overwhelming amount of information (Bruner, 1957; Hamilton and Trolier, 1986; Liberman et al., 2017; Macrae and Bodenhausen, 2000). For example, if we approach someone from a friendly group, we may believe it is likely that this person will be cooperative and trustworthy, rather than threatening (Fiske et al., 2002). Social categorization processes form the basis of how different groups and their members are perceived and evaluated in everyday social cognition. Yet this ability comes with significant drawbacks. As we simplify our surroundings through categorization we minimize the perceived differences between members of the same group (Brewer, 1988; Ostrom and Sedikides, 1992). At a minimum this leads to a loss of individual differentiation and stereotyping, but in more extreme cases this can result in xenophobia (Rydgren, 2004) and dehumanization (Haslam, 2006), for instance.

Given the ubiquitous nature of this phenomenon (Jylhä et al., 2022; Krott et al., 2018; Markowitz et al., 2021; Martherus et al., 2021), one may ask whether the neural representations we develop for groups are encoded in the brain as stable representations in memory, making them more difficult to avoid, or whether these are fleeting concepts, only temporarily constructed by the brain. The stable representation of social groups, if it indeed exists, is still poorly understood in current research. In this exploratory fMRI study, we examine whether repeated exposure to real-world social groups evokes repetition suppression, a neural signature often taken to reflect a stable memory-based representation, and whether such effects vary across different kinds of groups.

Many aspects of social information are stored in stable memory representations. For instance, personality traits, such as ‘friendly’ or ‘annoying’, tend to be represented and encoded in the medial prefrontal cortex (mPFC) (Arbula et al., 2021; Ma et al., 2014; Ma et al., 2016; Van Overwalle, 2009). Similarly, representations about oneself are activated and encoded in the same area (Heleven and Van Overwalle, 2018, 2019b), as are representations about known or unknown other individuals (Heleven et al., 2018; Heleven and Van Overwalle, 2018, 2019a). Even the conceptual knowledge we have about groups, in essence their stereotypes, also appear to rely on stable representations in the mPFC (Delplanque et al., 2019) including the lower ventral part of the mPFC (Kobayashi et al., 2022).

Yet, stable representations of the social groups themselves remain elusive. Group-related processes have been associated with a wide range of brain areas, varying by task and context. Although most of this research does not directly address whether groups are encoded as stable memory representations, this literature may indicate potential areas that support stable encoding functions. Molenberghs (2013) found a relationship between the process of group categorization and the mPFC, the anterior cingulate cortex, the temporoparietal junction (TPJ), and the precuneus. Other studies show that the dorsal mPFC is tuned to the processing of novel groups, while the ventral mPFC is preferentially activated for familiar ingroups as opposed to outgroups (Cikara and Van Bavel, 2014; Holbrook et al., 2016; Molenberghs, 2013; Sellaro et al., 2015; Shkurko, 2013). Outgroups also evoke greater activity in the anterior insula, possibly reflecting increased emotional salience or vigilance (Merritt et al., 2021). In addition to medial prefrontal and executive control regions, posterior temporal areas such as the posterior superior temporal sulcus (pSTS) have been consistently implicated in social-cognitive processing. The pSTS is commonly associated with the identification of biological (e.g., human) action, supporting the interpretation of others’ actions, intentions, and socially relevant contextual information (Isik et al., 2017; Schurz et al., 2020; Van Overwalle and Baetens, 2009). Rather than encoding stable trait-like representations per se, this region is thought to support the integration of dynamic social cues and the inference of meaning in context.

Yet, the question of stability of group membership is critical, because if it exists, it makes group identification more likely and hence may more easily recruit group stereotyping and biases (Brewer, 1988, 2007). For instance, ingroup members, whether defined by ethnicity (Guassi Moreira et al., 2017), or even regional dialect (Bestelmeyer et al., 2015), can evoke stronger bilateral amygdala responses, reflecting heightened emotional sensitivity (Merritt et al., 2021). While this may promote prosocial behaviors within the group, it also supports selective empathy (Bucchioni et al., 2015; Cikara and Van Bavel, 2014) and even dehumanization of outsiders (Harris and Fiske, 2006).

Of the brain areas discussed so far, it is unclear which, if any, may be responsible for the stable neural representation of groups. A widely used method to localize such representations is repetition suppression (Barron et al., 2016; Henson and Rugg, 2003; Miller et al., 1996). This approach capitalizes on a neural phenomenon whereby repeated exposure to the same stimulus leads to a decreased neural responsiveness in regions encoding that information. By systematically repeating a specific type of information (e.g., traits, individuals, social groups), researchers can identify areas that show decreased activity. This in turn is commonly interpreted as evidence that the brain has stored a stable representation of that information (Barron et al., 2016; Yee et al., 2010).

While repetition suppression is often treated as a proxy for memory-based encoding, alternative explanations have been put forward. Notably, in predictive coding frameworks, suppression may reflect a reduction in prediction error rather than memory adaptation (Barron et al., 2016). However, these two processes are not necessarily mutually exclusive. Even within a predictive coding view, a reduction in the prediction error signal is only meaningful if the neural population responsible encodes relevant information, or some aspect of it. What remains unclear is the durability of this encoding. Repetition suppression may reflect stable encoding in the context of a single experiment, but whether such stability extends beyond that timescale remains unknown.

There have been only a few studies using repetition suppression to identify stable memory representations of groups. In one study, Lau and Cikara (2017) investigated group representations by identifying participants with a party affiliation (Democratic or Republican) and assigning them to arbitrary groups (Eagles or Rattlers). Participants read about pairs of individuals belonging to identical ingroups (e.g., Eagles–Eagles), different ingroups (e.g., Eagles–Democrats), or mixed ingroup–outgroup pairs (e.g., Eagles–Rattlers). For each pair, the participants had to count the number of ingroup members they had detected. Changes in the fMRI signal from one half of the pair to the next were monitored to detect repetition suppression. Results showed that identical and different ingroup pairings lead to suppression in executive control areas (Yeo et al., 2011), including the superior parietal lobule, dorsolateral prefrontal cortex (dlPFC), and additionally the middle temporal gyrus (MTG). According to the authors, instead of the mPFC which relates to similarity of the groups with the self, these areas mainly reflect domain-general executive control processes possibly related to the functional significance of groups for the self (e.g., good or bad for me). However, a potential limitation to this study is related to the ostensible task instructions. By asking participants to count group members, executive control areas could be involved for this cognitive task rather than for social impressions. In addition, the authors only manipulated the suppression of ingroups and not of outgroups, which is surprising since outgroups are typically viewed as more monolithic and less diverse than ingroups, and thus could lead to stronger suppression effects (Quattrone and Jones, 1980). This opens the possibility that participants may have shown suppression by thinking of specific ingroup exemplars, rather than the ingroup as a whole.

Using a similar suppression task, Delplanque et al. (2019) presented pairs of either similar or dissimilar professional groups (e.g., police officer, train conductor, etc.) while participants had to judge the typicality of their behavior (e.g., “makes an arrest”, “scans a ticket”, etc.). Results showed repetition suppression for similar groups in the posterior cingulate cortex. However, a limitation of this study lies in the selection of the professional groups without any guidance of a theoretical framework. Moreover, it is unknown to what extent the participants in this study experienced a sense of belonging or emotionality with these groups. Therefore, further research is needed using a more systematic framework, in which real-world group exemplars are defined along meaningful social dimensions.

Social perception research has long emphasized that impressions of others emerge through an interplay between category-based processing and more individuated information (Fiske and Neuberg, 1990). One promising framework to operationalize this interplay is the Stereotype Content Model (SCM) developed by Fiske et al. (2002). This model delineates groups using two dimensions: warmth and competence. Warmth is the extent to which we feel positive emotions in response to a group, whereas competence is our subjective assessment of the capabilities of a group. Consequently, groups can be roughly divided into four distinct categories. Groups high in both warmth and competence (e.g., sports champions) are usually our ingroups or close allies, and usually evoke feelings of pride. On the other hand, groups which are considered low in both warmth and competence (e.g., drug addicts) are typically thought of as outgroups, and are associated with feelings of disgust. The two remaining categories evoke more mixed feelings. Groups high in warmth, but low in competence (e.g., disabled people) often induce feelings of pity, while conversely, low warmth and high competence groups (e.g., affluent people) induce envy. The advantage of this model is that it takes into account qualitative differences in our perception of specific categories of groups (Cuddy et al., 2008; Fiske et al., 2002). It expands the binary in- vs. outgroup distinction into a more nuanced and realistic typology. Moreover, the SCM has been extended through the Behaviors from Intergroup Affect and Stereotypes (BIAS) map, which links these stereotype dimensions to predictable behavioral tendencies toward social groups (Cuddy et al., 2007). For example, groups perceived as high in warmth tend to elicit helping and cooperative behaviors, whereas groups perceived as low in warmth may elicit avoidance or harm. This extension is relevant to the current study, as it illustrates how abstract stereotype dimensions are translated into concrete expectations about group-related actions. Importantly, the SCM distinguishes not only between positive and negative evaluations, but also between groups that are consistently high or low on both dimensions and those that combine high levels on one dimension with low levels on the other (Cuddy et al., 2007; Fiske et al., 2002). These latter ‘mixed’ groups are characterized by ambivalent evaluations (e.g., envy or pity), reflecting the coexistence of both positive and negative social inferences. In contrast, groups that are uniformly high or low on both dimensions tend to elicit more consistent and less ambivalent responses.

The SCM’s core dimensions have been replicated across cultures and social contexts, even though the specific group exemplars may vary with context (Cuddy et al., 2011; Durante et al., 2013; Fiske, 2018; Fiske et al., 2007; Grigoryev et al., 2019). Even though the model introduces a more detailed view of social categories, there is some evidence to suggest there is a substantial difference between the disgust-category (low in warmth and competence) and the other three types of groups. Harris and Fiske (2006) showed that only the disgust-category failed to evoke a response in the mPFC, an area vital to social cognition. This has led the authors to conclude that groups scoring low on both warmth and competence may be perceived as less than human. Yang et al. (2020) found a similar pattern of activity when monitoring the brain’s response to the four categories through an electroencephalogram (EEG). They observed that participants were slower to detect incongruent pairs of groups when the target belonged to the disgust-category. Moreover, congruent pairs of groups from the disgust-category caused a larger P2 spike, an EEG component which has been associated with stereotype processing (Dickter and Bartholow, 2007; Yang et al., 2020). The four categories also appear to have differential effects on social optimism (i.e., the expectation of a desirable future) and brain activation. Dricu et al. (2020) found that groups belonging to the disgust-category (low warmth/low competence) were typically associated with lower optimism, whereas the reverse was true for the pride-category (high warmth/high competence) and pity-category (high warmth/low competence). The disgust- and envy-categories (both low in warmth) were associated with other-related social areas, including the right anterior temporal lobe, and the TPJ. In addition, the disgust-category also recruited the anterior insula, inferior frontal cortex, and dlPFC (Dricu et al., 2020). The pride-category (high warmth/high competence; likely reflecting mainly the ingroup) was associated with self-related areas, including the ventral mPFC, precuneus, and posterior cingulate cortex. The pity-category (high warmth/low competence) was associated with sensory-related areas (Yeo et al., 2011), including the posterior insula and somatosensory cortex, and additionally the middle cingulate cortex.

In the current study, we used the SCM to select four representative group exemplar s at the extreme of each of the four SCM quadrants. That is, groups located near the point that was as far as possible from the other corners of the quadrants, to maximize conceptual contrast between categories. Consequently, there were no groups representing the midpoint of these dimensions. At each trial, participants viewed two brief sentences. The first sentence was a neutral prime description about one group followed by a second sentence consisting of a stereotypical target description referring either to the same group (repeated condition) or to a different group (non-repeated condition). After each trial, participants judged the typicality of the last target description relative to the group. This required participants to actively evaluate the relationship between the sentence and the social group, thereby encouraging the identification and retrieval of group-related knowledge during sentence processing. An example of a repeated group pair is the sentence “Military people sense a refreshing breeze” followed by the sentence “Military people are proud of their nation.” This paradigm allowed us to test whether the brain represents *group identity* in a stable fashion. Although the sentences in the prime (neutral) and target (stereotypical) sentences differed, the same social group was referenced across both sentences. Thus, repetition was defined at the level of group identity rather than repeated linguistic or perceptual content. If activation is suppressed after presenting the same group in the second sentence, this is indicative of stable group representation in memory. Lack of suppression would suggest an absence of stable group representation detectable by this method, and suggests that the group is represented more transiently, perhaps constructed *ad hoc* rather than retrieved from memory. This approach follows previous repetition suppression studies in social neuroscience that varied surface descriptions while repeating the identity of individuals (Heleven and Van Overwalle, 2019a), rather than groups.

Based on prior literature on the SCM, we expected to find similar areas to be activated for different types of groups. In line with Harris and Fiske (2006), we hypothesized that the mPFC would be associated with all categories, except for the disgust (low warmth, low competence) category. This is partially corroborated by Lau and Cikara (2017), who found the mPFC for ingroups, as well as the superior parietal lobule and MTG. As noted earlier, Dricu et al. (2020) further found that the disgust- and envy-categories were associated with other-related social areas, including the right anterior temporal lobe and TPJ, while the pity-category was associated with sensory-related areas, including the posterior insula and somatosensory cortex. Note that this study did not involve a repetition paradigm, but the results serve to provide a selective number of areas as likely candidates for stable representations. In addition, the posterior cingulate cortex has already been associated with the representation of groups by repetition suppression (Delplanque et al., 2019) and was therefore also considered a candidate region. To summarize, this study examined whether socially meaningful group categories display stable representations in memory, rather than being constructed entirely ad hoc. More specifically, we tested whether groups located in different SCM quadrants would elicit repetition suppression in social-cognitive brain regions previously implicated in person and group representation and processing. By doing so, the present study seeks to contribute to a better understanding of how socially shared group knowledge may be neurally organized.

## Method

### Participants

Participants consisted of 30 right-handed, neurologically healthy individuals (18 women) between the ages of 18 and 30 (*M* = 23.6), recruited from the university’s student population. All participants were Dutch speaking with normal, or corrected to normal vision. Due to both budgetary and practical constraints related to task length and participant fatigue, the experimental conditions were split between two participant groups, each consisting of 15 randomly assigned people. Each participant was exposed to two of the four group categories, either High Competence/Low Warmth and Low Competence/High Warmth (version A), or High Warmth/High Competence and Low Warmth/Low Competence (version B). This allowed us to present a sufficient number of repetition trials per condition without exceeding the total task duration of 45 min. To estimate the smallest effect size our study was capable of detecting, we conducted a sensitivity analysis using G*Power 3.1 (Faul et al., 2007). For the within-subject comparisons (prime vs. target), with a sample size of *N* = 15, *α* = 0.05 (one-tailed), and power = 0.80, the minimum detectable effect size was Cohen’s *d* = 0.76. This indicates that our design was sufficiently powered to detect large within-subject effects, but not small or moderate ones. For between-subject comparisons, the detectable effect size was *d* = 1.05, highlighting the limitations for cross-group inference.

The study protocol was approved by the Medical Ethics Committee at the University Hospital of Ghent, where the study was conducted, and the Free University of Brussels. Informed consent was acquired from the participants. In exchange for their collaboration, participants were compensated with 20 euros, an image of their brain, and traveling expenses.

### Stimulus materials

**Figure 1:**
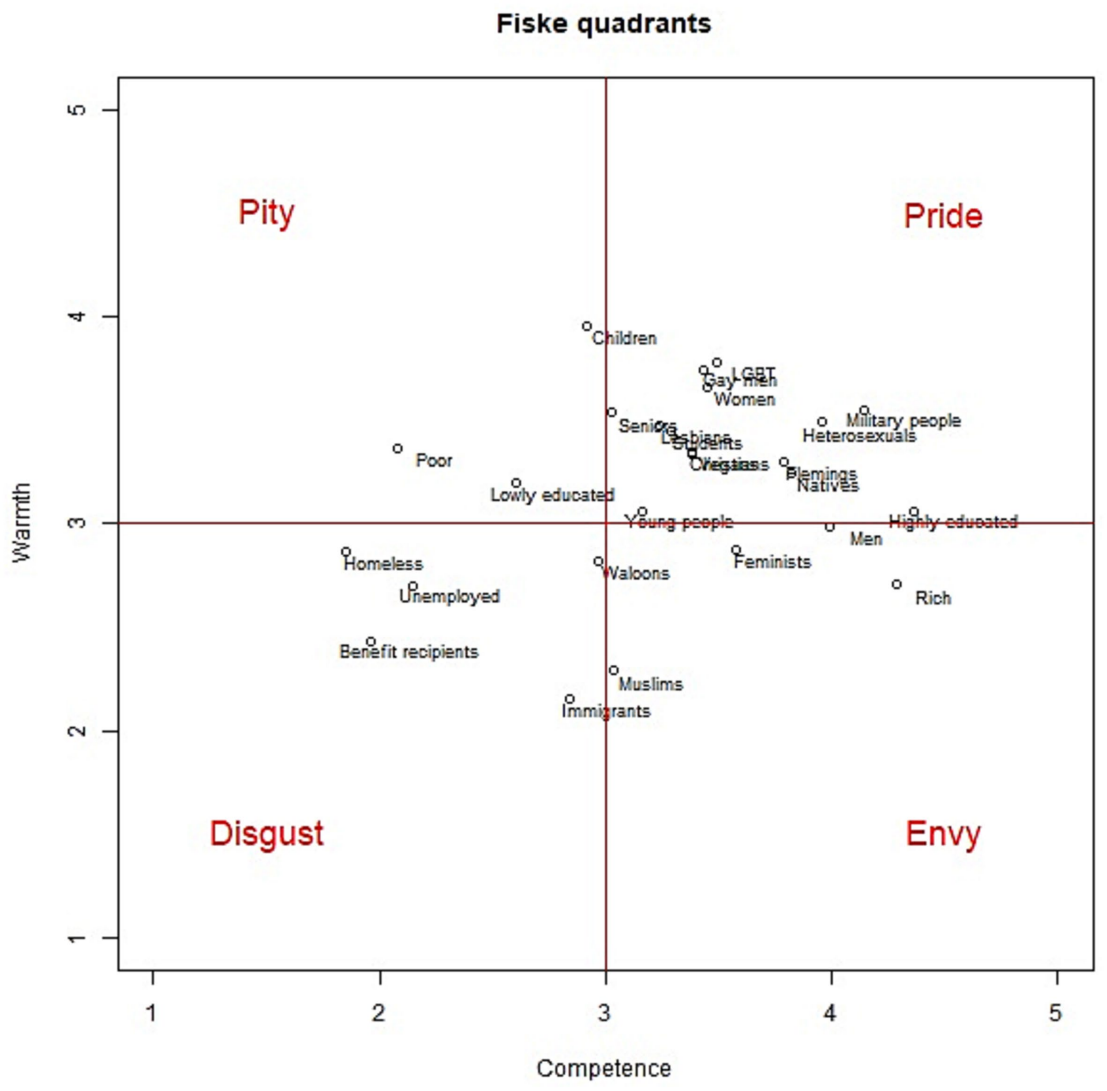
Dispersion of social groups according to pilot study 2 across the SCM quadrants. The *x*-axis shows a group’s competence. The *y*-axis shows a group’s warmth. The red lines are drawn at the mid-point of each axis to divide the graph into the four SCM quadrants. Each quadrant is labeled by the emotion generally experienced toward each group (Fiske et al., 2002).

In order to identify one group for each of the four SCM quadrants, several pilot studies were conducted. In a first study (*N* = 44), using an open question translated to Dutch from Fiske et al. (2002), we asked the participants which social groups currently defined society. Participants entered 8–16 groups that were then compiled and compared. The most common answers were used in the subsequent pilot study. In the second pilot (*N* = 30) the warmth and competence of 26 different groups was ascertained using a questionnaire translated to Dutch, also from Fiske et al. (2002). Warmth and competence were then plotted and the most extreme group exemplar, defined as most distant from the midpoint (3) of the 5-point in the expected direction on both warmth and competence, was selected for each of the four resulting quadrants (Figure 1). The group highest in both warmth (*M* = 3.55, SD = 0.73, *t*(29) = 4.11, *p* < 0.001) and competence (*M* = 4.15, SD = 0.46, *t*(29) = 13.55, *p* < 0.001) were Military People (in Dutch “militairen,” a generic term referring to any member of the military regardless of branch, such as navy or air force), the group lowest in both warmth (*M* = 2.39, SD = 0.61, *t*(29) = −5.42, *p* < 0.001) and competence (*M* = 2.07, SD = 0.55, *t*(29) = −9.35, *p* < 0.001) were Welfare Recipients (in Dutch a derogatory term was used: “doppers,” a label referring to individuals perceived as exploiting unemployment benefits), the group highest in warmth (*M* = 3.42, SD = 0.81, *t*(29) = 2.81, *p* = 0.009) and lowest in competence (*M* = 2.15, SD = 0.45, *t*(29) = −10.29, *p* < 0.001) were the Poor (in Dutch “armen”), and the group lowest in warmth (*M* = 2.66, SD = 0.70, *t*(29) = −2.66, *p* = 0.012) and highest in competence (*M* = 4.25, SD = 0.43, *t*(29) = 15.86, *p* < 0.001) were the Rich (in Dutch “rijken”). Participants were recruited from a university student population and were not selected based on membership in any of these social categories. The stimuli were intended to represent culturally shared stereotype categories associated with the warmth and competence dimensions of the SCM rather than participants’ personal group memberships. For each of these groups sentences were constructed describing either neutral or group-descriptive sentences (examples in Table 1). These sentences could include actions, but also common feelings, or experiences of the respective group. Therefore, sentences included both behavioral and affective content, reflecting the variety of ways in which social stereotypes are expressed in everyday language. Importantly, these different types of descriptions were distributed across the stimulus set and were not systematically associated with any specific group category. An initial pool of sentences was generated by the researchers based on commonly shared stereotypes and media portrayals associated with each social group. Typical sentences were evaluated in a third pilot study (*N* = 193), in which participants rated how stereotypical the group-description was relative to the group with a number ranging from 1 to 5. Sentences with a median score of at least 3 (moderately stereotypical) were selected for the main study. No additional ranking was applied; the threshold ensured that all selected sentences were perceived as at least moderately stereotypical of the intended group, while also preserving a sufficiently large and varied stimulus set for the experimental design. Neutral sentences were based on stimuli from two similar studies, but which investigated individuals rather than groups (Heleven et al., 2018; Heleven and Van Overwalle, 2015). In these studies, participants were likewise recruited from student populations comparable to the present sample. These sentences were used as neutral controls, scoring an average of 4 on a 7-point applicability scale, since they contained no group-related information and had previously been validated in comparable paradigms. On average sentences were 6.5 words long and varied between a minimum of 4 words and a maximum of 13 words.

**Table 1:** Overview of the conditions and sample sentences.

Condition	Prime sentence	Target sentence
Version A		
Military + Military (HH/HH)	Military people sense a refreshing breeze.	Military people are proud of their nation.
Military + Welfare (HH/LL)	Military people sense a refreshing breeze.	Welfare Recipients only pretend to look for a job.
Welfare + Welfare (LL/LL)	Welfare Recipients hear a quiet sound.	Welfare Recipients only pretend to look for a job.
Welfare + Military (LL/HH)	Welfare Recipients hear a quiet sound.	Military people are proud of their nation.
Singleton (–/HH)	—	Military people sense a refreshing breeze.
Version B		
Rich + Rich (LH/LH)	The rich hear a noise in the distance.	The rich often attend extravagant parties.
Rich + Poor (LH/HL)	The rich hear a noise in the distance.	The poor cannot even afford a car.
Poor + Poor (HL/HL)	The poor observe a few trees.	The poor cannot even afford a car.
Poor + Rich (HL/LH)	The poor observe a few trees.	The rich often attend extravagant parties.
Singleton (–/LH)	—	The rich hear a noise in the distance.

L = low on a dimension, H = high on a dimension; the 1st position identifies the warmth dimension, while the 2nd position identifies the competence dimension (e.g., HL = high warmth, low competence). In each version of the experiment half of the singleton consisted of the first group (e.g., military people) and the other half of the second group (e.g., welfare Recipients).

### Procedure

Before the experiment began participants received both written and oral instructions. They were told they would read sentences describing some social groups, and would have to indicate how typical they thought the description was for the group. Note that this judgment concerned the typicality of the description for the group, not the group itself. Participants had the opportunity to familiarize themselves with the task during a number of practice trials. Each trial began with the question “typical?” appearing at the center of the screen for 2 s to keep the participant focused on the task. This was followed by a fixation cross with a variable duration jittered between 0 and 2,000 ms randomly drawn from a uniform distribution. Afterwards the prime sentence was presented for 5,500 ms. This sentence was followed by another jittered fixation cross (0–2,000 ms) and the target sentence (5,500 ms). The participant then scored the most recent sentence (i.e., the target) with a typicality score: 1 = *very atypical*, 2 = *rather atypical*, 3 = *rather typical*, and 4 = *very typical*. Typicality ratings were not collected during prime presentation to avoid interrupting the repetition suppression effect, which requires prime and target to follow immediately without intervening processes. This design does not exclude the possibility that implicit typicality evaluation of the primes took place, as part of a continuous process of group evaluation. Because this process might have occurred across all conditions, we surmise that it contributed only to general, non-systematic variance rather than to condition-specific noise.

In order to have sufficient experimental trials per social group and condition, as noted earlier, the experiment was divided into two versions, called versions A and B. These versions were designed to be orthogonal in terms of stereotype dimensions, such that each participant encountered two groups that differed on both warmth and competence. This ensured that any observed repetition suppression would reflect a response to the full group profile, rather than repetition of only one stereotype dimension. It also reduced conceptual overlap within participants, supporting clearer interpretation of condition-specific neural effects.

Each version of the experiment consisted of five conditions. In version A of the experiment, participants read sentences describing Military People or Welfare Recipients. In the identical Military + Military condition both prime and target sentences revolved around Military People (high warmth, high competence), and in the identical Welfare + Welfare condition both sentences described Welfare Recipients (low warmth, low competence). There were also two mixed conditions (Military + Welfare and Welfare + Military) in which both groups were presented as either the prime or target. Finally there was a Singleton condition in which a target sentence was presented without a prime. This ensured that participants would not expect to respond only after every second sentence, thereby maintaining attention and consideration of group identity on each trial. Half of the sentences in this condition described Welfare Receiver and the other half described Military People. Across these conditions the sentences were either typical or neutral, as indicated by the pilot studies. Prime and singleton sentences were always neutral, while target sentences were always typical. In doing so, only the social group identity was repeated; the typicality of the sentences was never repeated and hence cannot in itself lead to repetition suppression.

In version B of the experiment sentences described either the Rich (low warmth, high competence) or the Poor (high warmth, low competence). As before there were two conditions with identical groups (Rich + Rich and Poor + Poor) and two mixed conditions (Rich + Poor and Poor + Rich). The Singleton condition consisted of target sentences describing either the Rich or the Poor. An overview of the conditions in both versions, including examples, can be found in Table 1. In both versions, there were 20 trials in each condition, for a total of 100 trials per version. All trials were presented in a random order for each participant. We counterbalanced the order of each condition between participants.

### Imaging procedure

Images were collected with a Siemens Magnetom Prisma fit scanner system (Siemens Medical Systems, Erlangen, Germany) using a 64-channel radiofrequency head coil. Stimuli were projected onto a screen at the end of the magnet bore that participants viewed by the way of a mirror mounted on the head coil. Stimulus presentation was controlled by E-prime 2.0 (Psychology Software Tools)^1^ running under Windows XP. Participants were placed head first and supine in the scanner bore. Participants were instructed not to move their heads to avoid motion artifacts. Foam cushions were placed within the head coil to minimize head movements. First, high-resolution anatomical images were acquired using a T1-weighted 3D MPRAGE sequence [TR = 2,250 ms, TE = 4.18 ms, TI = 900 ms, acquisition matrix = 256 × 256 × 176, sagittal FOV = 256 mm^3^, flip angle = 9°, voxel size = 1 × 1 × 1 mm]. Second, whole-brain functional images were collected in a single run using a T2*-weighted gradient echo sequence, sensitive to BOLD contrast (TR = 1,000 ms, TE = 31 ms, image matrix = 64 × 64, FOV = 210 mm, flip angle = 90°, slice thickness = 4.0 mm, distance factor = 10%, voxel size = 3.5 × 3.5 × 4 mm^3^, 35 axial slices).

### Image processing

The fMRI data were preprocessed using SPM12 (version 2; Wellcome Department of Cognitive Neurology, London, UK). Data were preprocessed to remove sources of noise and artifacts. Functional data were corrected for differences in acquisition time between slices for each whole-brain volume, realigned to correct for head movement, and co-registered with each participant’s anatomical data. The functional data were then transformed into a standard anatomical space (2 mm isotropic voxels) based on the ICBM152 brain template (Montreal Neurological Institute). Normalized data were then spatially smoothed (6 mm full-width at half-maximum, FWHM) using a Gaussian Kernel. Finally, the preprocessed data were examined using the Artifact Detection software package (ART),^2^^,^^3^ for excessive motion artifacts and for correlations between motion and experimental design, and between global mean signal and experimental design. Outliers were identified in the temporal differences series by assessing between-scan differences (Z-threshold: 3.0 mm, scan to scan movement threshold: 0.5 mm; rotation threshold: 0.02 radians). These outliers were controlled for in the analyses by including a single regressor for each outlier. No correlations between motion and experimental design or global signal and experimental design were identified. Six directions of motion parameters from the realignment step as well as outlier time points (defined by ART) were included as nuisance regressors. We used a default high-pass filter of 128 s and serial correlations were accounted for by the default auto-regressive AR(1) model.

### Statistical analysis

#### Behavioral analysis

We used two linear mixed-effects models to examine the effects of experimental condition on reaction times and typicality ratings. In both models, condition was included as a fixed effect, and participants were included as random intercepts with random slopes for condition to account for individual variability. Results are reported using a type III Wald chi square test for the fixed effects of condition.

#### fMRI analysis

Analysis of the fMRI data at the first (single participant) level were conducted using the general linear model of SPM12. The event-related design was modeled with two regressors for each condition (one for each prime and target sentence; there was only one regressor for the singleton condition), time locked at the presentation of the prime and target sentences and convolved with a canonical hemodynamic response function with event duration set to 0 for all conditions. This reflects the assumption that the key social-cognitive processing occurs rapidly following stimulus presentation, which is consistent with EEG evidence suggesting that both implicit and explicit social-cognitive processes typically unfold within a few hundred milliseconds rather than across the full duration of overt responding (Van Overwalle and Vandekerckhove, 2013). Six motion parameters from the realignment as well as outlier time points (identified by ART) were included as nuisance regressors. The response of the participant was not modeled separately.

For the group (second level) analyses, we conducted a full factorial whole-brain analysis of variance (ANOVA) with conditions as within-participants factor using a cluster-forming voxel-wise statistical threshold of *p* ≤ 0.001 (uncorrected) with a minimum cluster extent of 10 voxels, and identified significant clusters at a cluster-wise threshold of *p* < 0.05 (family wise error, FWE, corrected). Simple repetition suppression effects were defined for each condition by prime > target contrasts. As in prior research (Delplanque et al., 2019), we also specified asymmetric interactions by directly comparing prime > target contrasts for different conditions; this allows to test prime > target contrasts for one condition while controlling the same contrast for another condition. For example, the prime > target contrast of the repeated Poor group (prime = the Poor, target = the Poor) received weights +1 and −3, respectively, indicating suppression, while the comparison control condition with the same target group (prime = the Rich, target = the Poor) received for the prime > target contrast weights +1 and +1 indicating no suppression; this comparison thus attempted to identify a region that uniquely showed suppression for the Poor group, while also ensuring that the activity in the control condition (with the same Poor target group) remained stable. Although providing better control, these interaction effects might actually overlook potential suppression effects due to the strong constraint on the *a priori* weights used (only +1 and −3) while other weights are also theoretically plausible.

To examine if the brain areas identified in the simple and interaction analyses showed the predicted repetition suppression pattern, we computed the percent signal change. Regions of interest (ROIs) were defined based on peak voxels from significant clusters in the whole-brain analysis. These ROIs were used solely for signal extraction to examine the *direction* of repetition effects (i.e., whether differences in the contrast stemmed from decreased activation during the target). We did not use the ROI analysis to establish statistical significance, but to characterize how the suppression pattern looks and to rule out alternative explanations (e.g., increased activation during primes). This is driven by the limitation that contrast and interaction analyses in fMRI do not guarantee that the hypothesized suppression patterns are correctly identified, and to our knowledge, no alternative statistical procedure to do so in fMRI analysis is currently available. Given that these ROI analyses are performed on regions defined from the same dataset, the resulting statistical tests are not independent of the selection procedure and therefore do not constitute additional inferential evidence for the presence of the effect (Kriegeskorte et al., 2009). Rather, these analyses serve to further characterize the structure and direction of the observed effects within the identified clusters. Accordingly, the reported ROI statistics should be interpreted as follow-up analyses that inform the nature of the signal pattern (e.g., whether it is consistent with suppression), rather than as independent confirmation or filtering of whole-brain results. We therefore report these tests for transparency and interpretability, without applying additional corrections for multiple comparisons, as they are not treated as independent confirmatory tests. This approach is exploratory and consistent with prior work (Heleven and Van Overwalle, 2015; Ma et al., 2014). We identified regions of interest (ROIs) as a sphere of 8 mm around the peak coordinates of the simple and interaction contrasts. We then extracted the percent signal change in these ROIs for each participant using the MarsBar toolbox.^4^ We calculated repetition indices for each condition, which were defined as the percentage signal change of target minus prime sentences for each condition. These data were further analyzed using a repeated measures ANOVA with a threshold of *p* < 0.05. In addition we used one-tailed t-tests, with a threshold of *p* < 0.05, to test our directional hypotheses of repetition suppression (i.e., lower BOLD response for repeated stimuli). This approach follows previous repetition suppression studies (Heleven and Van Overwalle, 2015; Ma et al., 2014) and reflects our *a priori* assumption that repeated exposure would lead to decreased neural activity rather than increases. Finally, to examine whether repetition effects were not influenced by unequal baseline activation, we compared PSC values between primes across conditions and between primes and the singleton using two-sided paired t-tests, as no directional differences were expected for these baseline checks.

## Results

### Behavioral results

Behavioral results were analyzed using linear mixed-effects models with condition as the fixed effect and participants as a random effect, applied separately to Versions A and B. Fixed effects of condition are reported using type III Wald chi square tests.

The conditions in version A of the experiment had a significant effect on reaction times, Wald *χ*^2^(4) = 312.5, *p* < 0.001, and typicality ratings, Wald *χ*^2^(4) = 1,287, *p* < 0.001. Trials during which participants had to score the sentences referring to Welfare Recipients elicited slower responses (*M* = 1,798 ms) than trials with Military People (*M* = 1,486 ms). Responses were slowest on the singleton trials (*M* = 3,016 ms). Post-hoc pairwise comparisons with Tukey correction showed that sentences referring to Welfare Recipients were significantly slower than those referring to Military People (*p* = 0.025) and singleton trials were significantly slower than all other conditions (all *p* < 0.001). The group-descriptive sentences of Military People received higher average typicality scores (*M* = 3.43) than those of Welfare Recipients (*M* = 2.59). Typicality scores on the singleton trials were the lowest (*M* = 2.45). As before, pairwise comparisons showed that sentences describing Military People were rated as significantly more typical than those describing Welfare Recipients (*p* < 0.001). Singleton trials received significantly lower typicality ratings than all other conditions (all *p* < 0.01).

The conditions of version B also had a significant effect on reaction times, Wald *χ*^2^(4) = 458.4, *p* < 0.001, and typicality ratings, Wald *χ*^2^(4) = 174.2, *p* < 0.001. There were no great differences in speed between the Poor (*M* = 1,614) or the Rich (*M* = 1,621). However, responses were much slower on the singleton trials (*M* = 3,447 ms). Post-hoc pairwise comparisons with Tukey correction confirmed that reaction times did not differ significantly between the group conditions (all *p* > 0.80), whereas singleton trials were significantly slower than all other conditions (all *p* < 0.001). Both Poor (*M* = 2.96) and Rich (*M* = 2.99) group-descriptive sentences received similar typicality scores. Typicality scores on singleton trials were again the lowest (*M* = 2.45). Pairwise comparisons confirm that typicality ratings did not differ significantly between the group conditions (all *p* > 0.75), while singleton trials received significantly lower typicality ratings than all other conditions (all *p* < 0.001).

These results confirm that generally participants perceived the descriptions selected by the pilot study as typical in both versions of the experiment, as intended.

### fMRI results

We used the same analysis of the fMRI data as in earlier studies using repetition suppression of social entities (Delplanque et al., 2019; Heleven and Van Overwalle, 2015, 2018, 2019b; Ma et al., 2014). First, we conducted a whole-brain random effects analysis contrasting simple prime > target trials and also computed asymmetric interaction effects to identify potential repetition effects. Second, we used a percent signal change analysis to exclude false positives from the previous analysis.

Whole-brain analysis. Analysis of the simple prime > target contrasts yielded significant repetition suppression effects in both versions of the experiment (Table 2). Welfare Recipients caused suppression in the rolandic operculum, and superior temporal gyrus; the Rich in the parahippocampal gyrus, middle cingulate cortex, insula, and intraparietal lobule; and the Poor in these last three areas in addition to the MTG. No areas were significantly associated with Military people. The analysis of the interaction effects yielded suppression only for the Poor-Poor (repetition) vs. Rich-Poor (control) interaction (see method section) which was observed in the left insula and intraparietal lobule (Table 2).

**Table 2:** Repetition suppression (prime > target contrast) effects from the whole brain analysis.

Anatomical label	*x*	*y*	*z*	Voxels	Max *t*
Simple prime > target contrasts					
Low warmth—low competence (welfare recipients; version A)					
L Rolandic Operculum	−42	−24	22	302	4.20°
L Supramarginal Gyrus	−60	−30	30		3.36°
L Superior Temporal Gyrus	−50	−34	20		3.53°
R Superior Temporal Gyrus	52	−32	20	342	4.19°
R SupraMarginal Gyrus	62	−32	28		3.21°
R SupraMarginal Gyrus	42	−32	28		2.86°
L Precentral Gyrus	−42	−18	56	551	4.06**
L Precentral Gyrus	−34	−18	48		3.94**
L Postcentral Gyrus	−26	−34	54		3.66**
High warmth—high competence (military; version A)					
—					
Low warmth—high competence (rich; version B)					
L ParaHippocampal Gyrus	−32	−40	−10	321	5.96°
L Middle Cingulate Cortex	−14	−32	42	269	5.93°
L Insula Lobe	−40	−14	−2	255	5.32°
L Heschl’s Gyrus	−36	−22	8		4.26°
R Calcarine Gyrus	18	−50	8	303	5.23°
R Precuneus	10	−62	24		3.24°
Intraparietal Lobule	48	−28	28	446	4.02*
R SupraMarginal Gyrus	56	−32	26		3.68*
R SupraMarginal Gyrus	62	−26	28		3.31*
High warmth—low competence (poor; version B)					
L Middle Occipital Gyrus	−40	−76	30	504	6.36**
L Middle Temporal Gyrus	−54	−58	4		4.00**
L Middle Occipital Gyrus	−38	−64	18		3.64**
L Middle Cingulate Cortex	−10	−32	44	1,383	5.69***
R Middle Cingulate Cortex	12	−24	40		4.94***
L Postcentral Gyrus	−38	−20	48		3.94***
L Insula Lobe	−40	−12	−2	424	5.22*
L Heschl’s Gyrus	−38	−22	10		4.30*
L Rolandic Operculum	−42	−18	18		3.68*
Intraparietal Lobule	48	−28	28	989	4.90***
R Superior Temporal Gyrus	54	−30	16		4.40***
R SupraMarginal Gyrus	60	−28	32		4.23***
R Middle Occipital Gyrus	48	−74	26	823	4.57***
R Middle Temporal Gyrus (pSTS) ^Suppr^	54	−56	6		4.55***
R Middle Temporal Gyrus	40	−52	16		4.50***
Interaction suppression effects					
High warmth—low competence vs. control (poor-poor repetition vs. rich-poor control, version B)					
L Insula Lobe	−40	−14	−2	424	5.12*
L Heschl’s Gyrus	−38	−20	6		4.00*
L Rolandic Operculum	−42	−20	20		3.69*
Intraparietal Lobule	48	−28	28	587	4.38**
R Superior Temporal Gyrus	54	−30	16		3.94**
R SupraMarginal Gyrus	60	−26	32		3.72**

Results when the same group is repeated in a prime-target pair, either alone (“simple”) or in comparison with another group (“interaction”). Coordinates refer to the MNI (Montreal Neurological Institute) stereotaxic space. Whole-brain analysis thresholded at *p* < 0.001, uncorrected with ≥ 10 voxels. Reported are clusters with *p* < 0.05, FWE cluster-corrected. The superscript ^Suppr^ refers to the percent signal change analysis indicating significant suppression for some groups in the percent signal analysis. L = left, R = right.

°*p* < 0.10, *p < 0.05, **p < 0.01, ***p < 0.001 (FWE peak corrected).

Region of interest analysis. Following the whole-brain analysis, we computed the percent signal change within all ROIs identified by the whole-brain analysis, using a sphere centered on all significant peaks and subpeaks from both simple and interaction contrasts (see Table 2) with a radius of 8 mm. This follow-up analysis was intended to evaluate whether the extracted signal pattern from the whole-brain analysis was consistent with a suppression profile, by comparing the percent signal change of prime and target under repeated vs. non-repeated conditions. In particular, a suppression index was derived by subtracting the percent signal change during the prime from that of the target and a pattern consistent with suppression was defined as a stronger decrease in the repeated compared to the non-repeated condition.

**Figure 2:**
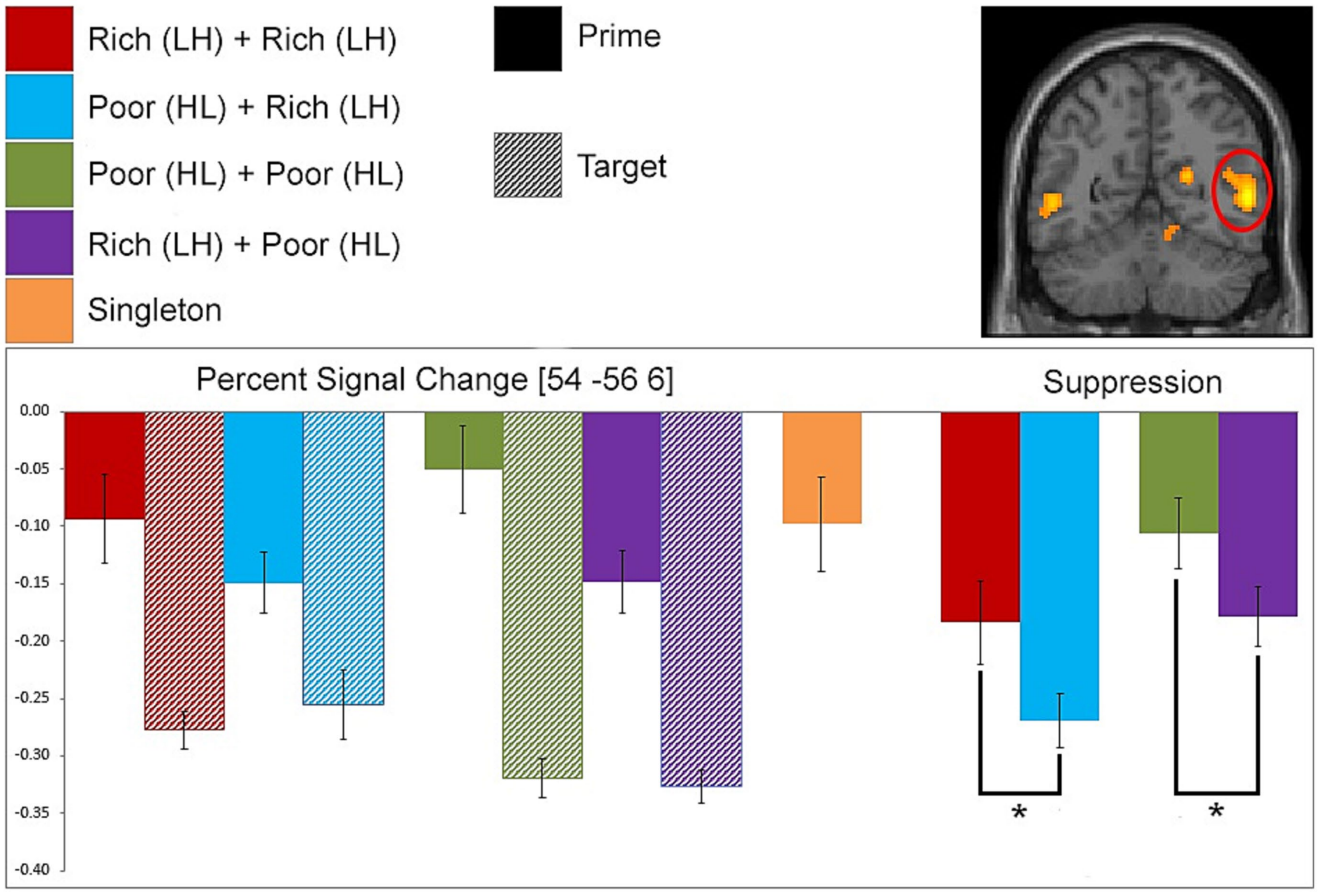
Percent signal change revealing significant group suppression. The left side of the panel shows the prime and target pairs, the right side shows the suppression index. Full colors refer to the prime, while dimmed colors refer to the target. Suppression is significant if the decrease of activation of a target group preceded by itself (repetition; red and green) is stronger than preceded by another group with opposite dimensions (non-repetition; blue and purple); *p < 0.05. As noted by the suppression index on the right of the figure, the red and blue conditions show that suppression is significantly higher for the Rich when it is preceded by another Rich trial, than by a Poor trial. The green and purple conditions show that suppression for the Poor is significantly greater when it is preceded by another Poor trial compared to a Rich trial. However, a comparison between primes shows that the prime of the Poor + Poor condition (full green) show significantly greater activity than those of the Poor + Rich (full blue) and Rich + Poor (full purple) conditions, *p* < 0.05 two-sided. The inset on the top right shows the same MTG/pSTS area (encircled) revealed by the whole-brain analysis, thresholded at *p* < 0.001 uncorrected, from which the signal change was extracted and indicated with its MNI coordinates.

Out of all the examined clusters, the extracted signal pattern in the right MTG was most consistent with a repetition suppression profile for both the Rich and Poor conditions (see Figure 2). Follow-up ANOVAs on the suppression indices yielded significant effects for the Rich, *F*(1, 14) = 5.226, *p* = 0.038, *η*_p_^2^ = 0.27, and the Poor conditions, *F*(1, 14) = 6.444, *p* = 0.024, *η*_p_^2^ = 0.32. The coordinates of this particular area (54–56 6) are also known in social neuroscience as the posterior superior temporal sulcus (pSTS; ±50–55 10; Van Overwalle and Baetens, 2009), and is closely located to the more superiorly located TPJ (±50–55 25; Van Overwalle and Baetens, 2009). No comparable suppression pattern was observed for either Military people or Welfare Recipients based on this method. Additional one-sided t-tests did not yield additional effects. As noted in the sensitivity analysis, the design was only powered to detect large effects (*d* ≥ 0.76). Null results, particularly in the Military and Welfare conditions, should therefore be interpreted with caution. Moreover, given the non-independence of the ROI definition and statistical testing, these results should be interpreted as characterizing the observed signal pattern rather than as independent confirmation of the effect.

To ensure that repetition effects were not caused by unequal baseline activity, we examined PSC values for prime sentences across conditions. None of the primes in any of the conditions differed significantly from the singleton trials (all two-sided *p* > 0.05). However, there was a slight upward deviation of the prime’s activity in the Poor + Poor condition compared to the Rich + Poor, *t*(14) = 2.14, *p* = 0.043, and Poor + Rich, *t*(14) = 2.14, *p* = 0.050, conditions. There were no other significant differences among the primes.

## Discussion

As an exploratory step toward identifying candidate brain regions involved in the neural representation of social groups, this study aimed to identify such regions using social groups that were drawn from the four quadrants of the SCM. Participants viewed group-descriptive sentences associated with each group in repeated and non-repeated pairings, allowing us to test for repetition suppression, used as a proxy measurement of stable memory representations. The whole-brain analysis revealed significant repetition suppression for three of the groups. Welfare Recipients caused suppression in the rolandic operculum and superior temporal gyrus. The Rich and Poor showed suppression in the parahippocampal gyrus, middle cingulate cortex, insula, and intraparietal lobule. Additionally the Poor also revealed suppression in the MTG. Further investigation of these results through a percent signal change analysis indicated a signal pattern consistent with repetition suppression for the Rich (low warmth, high competence) and Poor (high warmth, low competence) in the right MTG, in particular the right pSTS. No such suppression patterns were observed for either Military people (high warmth, high competence), or Welfare Recipients (low warmth, low competence).

Taken together, these findings only partially align with our hypotheses derived from prior work on the Stereotype Content Model. Based on earlier literature, we expected similar brain regions to be involved in representing different social groups, with the mPFC playing a central role for most categories except those belonging to the Welfare Recipients. In the present study, however, reliable repetition suppression was not observed in the mPFC or other candidate regions identified in previous studies, but rather in the right pSTS for the Rich (high on competence) and Poor (high on warmth), while no clear suppression pattern was found for the Military, who elicit the highest warmth and competence. In line with earlier research (Harris and Fiske, 2006), we did not find a stable representation for Welfare Recipients (low on both dimensions).

These results should be treated with caution, as some significant differences were observed among the primes. Activity for the prime in the Poor + Poor condition was higher than for the primes in the Rich + Poor and Poor + Rich conditions. The reason for this increased activity is not immediately clear. It is unlikely to stem from differences in content, since prime activity in the Poor + Rich condition was significantly lower than in the Poor + Poor condition, despite having identical primes. The apparent differences between similar primes could stem from the fact that the hemodynamic response unfolds over several seconds, so that residual activity from the preceding prime trial can partly overlap with the following target trial. This temporal overlap may introduce shared variance between primes and targets in the same areas across conditions, leading to slight prime increases (Henson, 2016). Given the average separation of 6.5 s between primes and targets in the present study, while the hemodynamic response typically peaks after about 5–7 s and gradually drops off until 20–30 s later, such overlap is possible. This overlap could lead to what appears as higher activation of the prime. An additional, though less plausible, possibility is that small prime differences reflect contextual or attentional fluctuations rather than stimulus properties. Repetition suppression is known to vary with attention, expectation, and recent trial history: when an event is anticipated or receives less attentional weight, its neural response is reduced even if the physical stimulus is identical (Larsson and Smith, 2012; Summerfield et al., 2008). Such top-down influences can therefore modulate baseline activation of the same group prime depending on the cognitive context in which it appears. Taken together, these considerations suggest that the observed pattern should perhaps not be interpreted as unequivocal evidence of classical repetition suppression. Rather, the findings are more cautiously interpreted as being compatible with the possibility that group-related information was processed in a relatively stable manner in this social-cognitive region, while acknowledging that differences in prime activation complicate this interpretation. The right temporal lobe was one of the hypothesized candidate brain areas selected from the fMRI activation study by Dricu et al. (2020), albeit for two different group categories related to low warmth and high or low competence. The MTG in particular was identified in the fMRI suppression study for ingroups by Lau and Cikara (2017). It is important to note that the particular area showing suppression in the present results is known for its social function, and typically referred to as the pSTS, closely located to the more superiorly located TPJ. It is part of the default mode network (Yeo et al., 2011), which supports social understanding of the mental state and traits of others, including other groups and their stereotypes (Molenberghs et al., 2016; Schurz et al., 2020). This region has been consistently implicated in the interpretation of others’ actions, intentions, and socially relevant contextual information (Isik et al., 2017; Schurz et al., 2020; Van Overwalle and Baetens, 2009). Rather than reflecting stable trait representations in the same sense as the mPFC, pSTS activity is often linked to the integration of dynamic social cues and the construction of meaning in context. In this light, the observed repetition effects may reflect not only the encoding of group identity, but also understanding the behavioral implications of that identity. This interpretation aligns with the present task, in which participants evaluated the typicality of group-related behaviors, a process that likely required mapping group identity onto expected actions.

Beyond the pSTS, the area most clearly displaying a repetition suppression pattern, the whole-brain analysis identified a broader network of regions. These map onto large-scale systems commonly implicated in social cognition. Research indicates that social understanding relies on distributed networks supporting contextual memory, affective evaluation, and attentional integration (Andrews-Hanna et al., 2010; Buckner et al., 2008; Corbetta and Shulman, 2002; Menon, 2011; Schurz et al., 2014; Shackman et al., 2011; Van Overwalle and Baetens, 2009). Within this framework, the parahippocampal gyrus may support the contextual retrieval of group-related knowledge, while the superior temporal gyrus forms part of a broader temporal system together with the pSTS involved in processing socially meaningful information. The insula and middle cingulate cortex are associated with salience and affective integration, reflecting the evaluation of socially relevant stimuli, whereas the intraparietal lobule contributes to the integration of human action and use of objects. The involvement of the rolandic operculum may reflect more basic sensorimotor or embodied aspects of social information processing.

While exploratory, this finding may have important implications for our understanding of how groups are cognitively represented and how these representations relate to the neural regions predicted by prior SCM research. It suggests that we may have stable representations of some social groups, but not of others. With stable group representations, we are able to draw largely from encoded history and experiences irrespective of the current context, potentially allowing for more consistent social inferences or responses. Without stable group representations, we may assemble a fluid representation from diverse memory traces upon encountering members of these groups across different contexts and situations which may prime distinct aspects of this fluid representation. This may result in more variable responses, perhaps reflecting differences in how individuals interpret or construct the social category in a given context. This distinction in response readiness across group types is not often emphasized in social psychology, but our results suggest it may warrant further investigation. However, the present findings do not clarify why certain groups appear to be represented, while others are constructed dynamically. This remains a question for future inquiry.

One tentative explanation for the presence of stable representations for the Poor and Rich groups is that these groups have mixed levels of warmth and competence (high on one and low on the other). This asymmetry may create ambiguity regarding their intentions or social value, prompting the need for more attentional resources to determine their functional relevance (e.g., threat or affiliation). This may prompt the brain to form stable representations to save resources in the long term. Conversely, groups that are clearly high or low on both SCM dimensions could allow quick responses without stable representation (as was the case for military people) if their functional relevance is more straightforward, but slower responses are also possible (as was the case for Welfare recipients) when there is (perhaps) little consensus among observers about their functionality. This interpretation is in line with Lau and Cikara (2017), who suggested that a stable group representation reflects a group’s functional significance, particularly whether they are “for” or “against” the self.

Beyond the present findings, the possibility that some social groups may show stable representations raises broader questions about the persistence and automaticity of social cognition. If certain group representations are more readily retrieved from memory, this may contribute to the rapid and often implicit activation of stereotypes in everyday perception. Such representations could, in principle, be more resistant to change, particularly if they are reinforced across repeated social experiences. At the same time, it is important to note that the present study does not directly assess the durability or malleability of these representations over time, and the observed effects should be interpreted within the experimental context.

There is a noteworthy discrepancy between the current findings and those of Lau and Cikara (2017), who found repetition suppression for ingroups in the mPFC. In the SCM, the high warmth and high competence quadrant is often most positively evaluated and can correspond with ingroups in many contexts (Fiske et al., 2002). While it is quite unlikely that military people constituted an ingroup in this study (participants were students at a regular university without any military ties, which is typical in Belgium), it did evoke the positive evaluations associated with this quadrant. The lack of personal ties with the Military group, however, may explain why we found no evidence of suppression in the mPFC, or any other candidate region associated with social cognition. Moreover, it is not immediately clear that the political ingroups used by Lau and Cikara (2017) would occupy the high warmth and high competence quadrant had they been viewed through the lens of the SCM. In general, political parties are often viewed with a measure of skepticism, even by their supporters, which could place them in one of the mixed quadrants. Research suggests that Americans often vote more *against* the opposing party than *for* their own, reflecting ambivalence rather than strong identification (Abramowitz and Webster, 2018). Furthermore, both U.S. political parties are perceived as elite institutions with significant wealth and power (La Raja and Schaffner, 2015), perhaps similar to our study’s high competence and low warmth category, the rich. Even minimal in- and outgroups may evoke mixed feelings through their novelty. While this remains speculative, it underscores the value the SCM could have to make such nuanced comparisons in future research.

Compared to other aspects of social cognition, the neural representation of groups remains substantially more elusive. Repetition suppression studies consistently demonstrate the mPFC’s role in the representation of social traits (Arbula et al., 2021; Li et al., 2021; Ma et al., 2014, 2016; Van Overwalle, 2009), including stereotypical traits of groups (Delplanque et al., 2019), the self (Heleven and Van Overwalle, 2018, 2019b), and individual others (Heleven et al., 2018; Heleven and Van Overwalle, 2018, 2019a), yet results become inconsistent at the level of the group (Delplanque et al., 2019; Lau and Cikara, 2017). Our own study was also largely unable to replicate suppression effects found in previous studies. While this will at least in part stem from methodological differences and statistical limitations, one plausible explanation is that groups are inherently more fluid. Unlike individual persons, who typically exhibit stable physical and psychological characteristics, group composition and identity can shift rapidly through changes in membership, leadership, or public perception. For example, Kotzur et al. (2019) demonstrated that groups can move across the SCM quadrants through exposure to individual group members. Participants who had higher exposure to asylum seekers tended to rate them as warmer compared to those with little exposure. This indicates that group perception, and by extension its neural representation, may be highly malleable. We suggest that the mPFC might perhaps encode stable trait dimensions, whereas group identity, which is defined by a conjunction of traits (i.e., warmth and competence in the present study), constitutes a broader and more context-dependent emergent abstraction.

Besides methodological differences, these inconsistencies may also reflect deeper conceptual ambiguity. Pietraszewski (2022) argues that the term “group” is often applied inconsistently across studies. His framework integrates many aspects such as perceived similarity, a shared label, sense of social identity, stereotyping, and an ingroup bias. The SCM captures some of these features, but it does not address all of them. For instance, it is possible that some groups (e.g., Military People) are perceived as more homogeneous than others (e.g., the Rich or the Poor), which might influence how easily a shared representation is formed. As such, use of SCM categories may limit comparability with studies that focus on more traditional ingroup-outgroup distinctions. These definitional ambiguities may help explain inconsistencies in the literature and suggest that group representation in the brain is more complex than current models allow.

As with any exploratory fMRI, study the results should be interpreted in light of certain methodological limitations. First, the sample size was modest and significantly limited the sensitivity of the study, especially for the detection of more subtle and intermediate effects. Second, the use of single exemplars for each of the SCM quadrants, while controlled, may not capture the diversity of perceptions typically associated with these categories. Adding to this, group selection was based on a separate pilot conducted in a comparable student sample, which supports the assumption of broadly shared representations. Still, individual differences in how participants in the main study perceive these groups were not assessed. Future research may benefit from collecting participant-specific ratings and incorporating them as covariates. Although all selected groups differed significantly from the midpoint of the SCM space in the expected direction on both warmth and competence, they were not equally differentiated across both dimensions. In particular, the contrast between groups was somewhat more pronounced for competence than for warmth. This asymmetry may have contributed to variation in the observed neural effects across categories. In addition, the group-descriptive sentences varied somewhat in whether they emphasized behavioral actions or affective characteristics. Prior research suggests that such differences may involve distinct levels of cognitive processing complexity (Taylor et al., 1978). Although these descriptions were selected through pilot testing to ensure comparable typicality across groups, such variation may have introduced additional noise into the neural responses and thereby reduced the likelihood of detecting repetition suppression effects. Moreover, the label used for Welfare Recipients (‘doppers’) carries a somewhat negative connotation in Dutch. While this is consistent with the evaluative profile typically associated with groups in the low warmth/low competence quadrant of the SCM, it remains possible that subtle differences in label connotation influenced participants’ responses to some extent. Third, because the sample was split into two versions (A and B), the lack of suppression for non-mixed SCM groups may stem from between-subject differences rather than stimulus properties. Although participants were randomly assigned and drawn from the same student population using the same task and scanning protocol (i.e., factors which reduce the likelihood of gross cohort differences), this does not entirely remove the inferential limitation of using a split sample. Furthermore, the SCM groups included in the two experimental versions differed in their thematic content. Version B contained economically defined categories (Rich and Poor), which may evoke particularly salient or culturally entrenched stereotypes. More specifically, it is possible that the observed effects for the Rich and Poor were driven not only by their mixed SCM profile, but also by their position along a salient socio-economic status dimension. If so, the apparent sensitivity of the pSTS to these categories may reflect the functional or social relevance of economic hierarchy, rather than mixed warmth and competence. In contrast, the groups in Version A (Military people and Welfare recipients) may be interpreted more variably across participants. For example, perceptions of Military people may depend strongly on (lack of) personal or familial connections, while attitudes toward Welfare recipients may be shaped by broader political beliefs. These factors may have introduced additional variability in how participants construed the groups, making it more difficult to conclude that stable neural representations emerge for some categories but not others based solely on the specific groups used in the present study. Fourth, while repetition suppression is widely used as a proxy for memory-based representation, its neural basis is not fully understood and may also reflect predictive coding or attentional mechanisms (Barron et al., 2016; Larsson and Smith, 2012). Moreover, because singleton trials differed from target trials in typicality, they only controlled for group identity (the main variable of interest) and not typicality of the full sentence. In addition, although the neutral control sentences were drawn from previously validated paradigms, their neutrality was not independently revalidated with respect to the four SCM groups. This may have introduced additional noise into the comparison, although the behavioral typicality ratings suggest that these neutral sentences were generally experienced as less group-typical than the stereotypical target descriptions. The observed repetition effects should therefore be interpreted with these limitations in mind. Fifth, our task design directed participant’s attention to group descriptions rather than explicit social categorization, which may have influenced the neural systems recruited. This consideration is also relevant for interpreting neural activity in regions associated with social cognition, which are located in the default mode network (DMN), such as the pSTS. The mentalizing subsystem of the DMN is reliably activated during social judgments and trait inferences (Andrews-Hanna et al., 2010; Schurz et al., 2014; Van Overwalle and Baetens, 2009). Hence, decreased activation after repetition of the same social information and judgment is most plausibly interpreted as repetition suppression. In a social context where these regions typically are more activated, it makes little sense to interpret this as repetition enhancement.

The present study suggests many avenues of future research. First, research may benefit from moving beyond the binary distinction between in- vs. outgroup to more fully capture how social groups are represented in the brain. Our findings suggest that the extent to which groups are represented in a stable fashion may depend on the type of group, with groups with mixed basic characteristics (i.e., Poor and Rich showing high vs. low warmth and low vs. high competence respectively) potentially showing more robust representation. The SCM offers a promising framework for parsing the differences between groups, but additional research is required to generalize these results using larger samples, varied exemplars within each quadrant, and converging methods. Future work may also benefit from modeling warmth and competence as continuous dimensions, rather than only as categorical quadrant labels, to examine whether neural effects scale with dimensional distance within the SCM space. Second, research should investigate whether the distinction between mixed and non-mixed groups reliably predicts neural representation and whether the pSTS (or related TPJ) consistently supports stable group representation. The reason for this representation also merits investigation. If groups are represented in the pSTS because mixed groups cause ambiguous functional relevance, then comparing ambiguous to non-ambiguous groups is vital. This could be achieved by having participants rate how important knowledge of group identity is when evaluating behavior and to include this score as a parametric modulator. Ideally, this would be studied using a within-subjects design. There are at least two avenues to achieve this without sacrificing significant statistical power. The first option is to present all conditions to each participant across multiple runs, thus preserving the number of trials and preventing exhaustion. However, this may introduce effects such as task reflection between sessions or changes in attentional state. These can be mitigated through standard procedures (e.g., counterbalancing run order, minimizing inter-run delays, maintaining task consistency). Alternatively, future studies could reduce the number of trials per condition and compensate with a larger sample size, trading within-subject reliability for increased generalizability and statistical power, although this comes with practical cost considerations. Third, research could also contrast regions associated with stable trait representation, such as the mPFC, with those sensitive to contextual meaning, such as the pSTS and TPJ. Presenting the same group across differing social contexts (e.g., cooperative vs. competitive) could reveal whether the pSTS flexibly adapts to situational relevance, while mPFC encodes more abstract, context-independent aspects of group knowledge. Fourth, to investigate some of the methodological caveats in the current study, future research could benefit from manipulating prime-target contingency. By parametrically varying the probability that a given target will follow a certain prime, researchers may be able to disentangle suppression due to repetition versus predictability. In the same vein, future studies might explore attentional modulation of suppression effects, for example by varying whether group identity is task-relevant (i.e., high-relevance), or not. Fifth, future research could investigate whether neural markers of repetition suppression are associated with the persistence of stereotypical beliefs, resistance to counter-stereotypical information, or the degree to which social categories are perceived as fixed versus flexible. Finally, a broader challenge for social neuroscience is to track how group representations emerge and evolve in real-world interactions. While tightly controlling repetition paradigms allow precise isolation of neural representations, future would could extend this approach using more naturalistic methods, such as movie viewing (Nastase et al., 2020), or interactive group tasks that capture ongoing social dynamics. These paradigms could benefit from the *a priori* regions of interest identified here (i.e., pSTS/TPJ). At the same time, laboratory-based manipulations can model these dynamics under controlled conditions. For instance, this could be done by gradually introducing new groups, varying group composition across trials, or changing functional relevance while holding behavioral content constant. Such designs may bridge the gap between localized repetition effects and the flexible, evolving representations of groups that characterize everyday social interaction.

## Conclusion

This study investigated whether social groups show evidence of relatively stable neural representations in memory using the Stereotype Content Model (SCM). We found a pattern fitting repetition suppression in a social area, the pSTS, suggesting stable representations in this area for groups with mixed levels of warmth and competence: the Poor (high warmth, low competence) and the Rich (low warmth, high competence). In contrast, no significant suppression effects were observed for non-mixed groups: the Military (high warmth, high competence) and Welfare Recipients (low warmth, low competence). These stable representations may facilitate less variable responses and could reflect the pSTS’s involvement in social group processes supported by the DMN. We propose that mixed groups evoke ambiguity, or uncertainty which may require stronger memory representations to quickly determine our functional relation to them (good vs. bad, or safe vs. unsafe). Non-mixed groups, by contrast, may be easier to appraise and may therefore rely more strongly on online or context-dependent construction. These results enrich our understanding of how the brain represents social groups, and highlights the interplay between stable and dynamic mechanisms in social categorization. Further research in this area could deepen our knowledge of group processing and its broader impact on social behavior and interaction.

## Data Availability

All requested (pseudonymized or anonymous) fMRI data are available upon request, excluding data that allow identifying individual participants. All manuals and code for processing the data is also available together with the data. Behavioral data, stimulus materials, pilot data, and its analyses are available via the Open Science Framework at https://osf.io/tdxyu.

## Footnotes

1
www.pstnet.com/eprime

2
http://web.mit.edu/swg/art/art.pdf

3
http://www.nitrc.org/projects/artifact_detect

4
http://marsbar.sourceforge.net

## References

[ref1] Abramowitz A. I. Webster S. W. (2018). Negative partisanship: why Americans dislike parties but behave like rabid partisans. Polit. Psychol. 39, 119–135. doi: 10.1111/pops.12479

[ref2] Andrews-Hanna J. R. Reidler J. S. Sepulcre J. Poulin R. Buckner R. L. (2010). Functional-anatomic fractionation of the brain’s default network. Neuron 65, 550–562. doi: 10.1016/j.neuron.2010.02.005, 20188659 PMC2848443

[ref3] Arbula S. Pisanu E. Rumiati R. I. (2021). Representation of social content in dorsomedial prefrontal cortex underlies individual differences in agreeableness trait. NeuroImage 235:118049. doi: 10.1016/j.neuroimage.2021.118049, 33848626

[ref4] Barron H. C. Garvert M. M. Behrens T. E. J. (2016). Repetition suppression: a means to index neural representations using BOLD? Philos. Trans. Roy. Soc. B Biol. Sci. 371:20150355. doi: 10.1098/rstb.2015.0355, 27574308 PMC5003856

[ref5] Bestelmeyer P. E. G. Belin P. Ladd D. R. (2015). A neural marker for social bias toward in-group accents. Cereb. Cortex 25, 3953–3961. doi: 10.1093/cercor/bhu282, 25452578 PMC4585525

[ref6] Brewer M. B. (1988). “A dual process model of impression formation,” in Advances in Social Cognition, eds. Wyer R. S. Jr Srull T. K. (Vol. 1) Hillsdale, NJ: Lawrence Erlbaum Associates, 1–36.

[ref7] Brewer M. B. (2007). “The social psychology of intergroup relations: social categorization, ingroup bias, and outgroup prejudice,” in Social Psychology: Handbook of Basic Principles, eds. Kruglanski A. W. Higgins E. T. (2nd ed.) New York, NY: Guilford Press. 695–715.

[ref8] Bruner J. (1957). On perceptual readiness. Psychol. Rev. 64, 123–152. doi: 10.1037/h0043805, 13420288

[ref9] Bucchioni G. Lelard T. Ahmaidi S. Godefroy O. Krystkowiak P. Mouras H. (2015). Do we feel the same empathy for loved and hated peers? PLoS One 10, 1–11. doi: 10.1371/journal.pone.0125871, 26024234 PMC4449017

[ref10] Buckner R. L. Andrews-Hanna J. R. Schacter D. L. (2008). The brain’s default network: anatomy, function, and relevance to disease. Ann. N. Y. Acad. Sci. 1124, 1–38. doi: 10.1196/annals.1440.011, 18400922

[ref11] Cikara M. Van Bavel J. J. (2014). The neuroscience of intergroup relations: an integrative review. Perspect. Psychol. Sci. 9, 245–274. doi: 10.1177/1745691614527464, 26173262

[ref12] Corbetta M. Shulman G. L. (2002). Control of goal-directed and stimulus-driven attention in the brain. Nat. Rev. Neurosci. 3, 201–215. doi: 10.1038/nrn755, 11994752

[ref13] Cuddy A. J. C. Fiske S. T. Glick P. (2007). The BIAS map: behaviors from intergroup affect and stereotypes. J. Pers. Soc. Psychol. 92, 631–648. doi: 10.1037/0022-3514.92.4.631, 17469949

[ref14] Cuddy A. J. C. Fiske S. T. Glick P. (2008). Warmth and competence as universal dimensions of social perception: the stereotype content model and the BIAS map. Adv. Exp. Soc. Psychol. 40, 61–149. doi: 10.1016/S0065-2601(07)00002-0

[ref15] Cuddy A. J. C. Glick P. Beninger A. (2011). The dynamics of warmth and competence judgments, and their outcomes in organizations. Res. Organ. Behav. 31, 73–98. doi: 10.1016/j.riob.2011.10.004

[ref16] Delplanque J. Heleven E. Van Overwalle F. (2019). Neural representations of groups and stereotypes using fMRI repetition suppression. Sci. Rep. 9:3190. doi: 10.1038/s41598-019-39859-y, 30816252 PMC6395704

[ref17] Dickter C. L. Bartholow B. D. (2007). Racial ingroup and outgroup attention biases revealed by event-related brain potentials. Soc. Cogn. Affect. Neurosci. 2, 189–198. doi: 10.1093/scan/nsm012, 18985140 PMC2569810

[ref18] Dricu M. Schüpbach L. Bristle M. Wiest R. Moser D. A. Aue T. (2020). Group membership dictates the neural correlates of social optimism biases. Sci. Rep. 10, 1139–1117. doi: 10.1038/s41598-020-58121-4, 31980697 PMC6981267

[ref19] Durante F. Fiske S. T. Kervyn N. Cuddy A. J. C. Akande D. Adetoun B. E. . (2013). Nations’ income inequality predicts ambivalence in stereotype content: how societies mind the gap. Br. J. Soc. Psychol. 52, 726–746. doi: 10.1111/bjso.12005.Nations, 23039178 PMC3855559

[ref20] Faul F. Erdfelder E. Lang A. G. Buchner A. (2007). G*power 3: a flexible statistical power analysis program for the social, behavioral, and biomedical sciences. Behav. Res. Methods 39, 175–191. doi: 10.3758/BF03193146, 17695343

[ref21] Fiske S. T. (2018). Stereotype content: warmth and competence endure. Curr. Dir. Psychol. Sci. 27, 67–73. doi: 10.1177/0963721417738825, 29755213 PMC5945217

[ref22] Fiske S. T. Cuddy A. J. C. Glick P. (2007). Universal dimensions of social cognition: warmth and competence. Trends Cogn. Sci. 11, 77–83. doi: 10.1016/j.tics.2006.11.005, 17188552

[ref23] Fiske S. T. Cuddy A. J. C. Glick P. Xu J. (2002). A model of (often mixed) stereotype content: competence and warmth respectively follow from perceived status and competition. J. Pers. Soc. Psychol. 82, 878–902. doi: 10.1037/0022-3514.82.6.878, 12051578

[ref24] Fiske S. T. Neuberg S. L. (1990). “A continuum of impression formation, from category-based to individuating processes: influences of information and motivation on attention and interpretation,” in Advances in Experimental Social Psychology, ed. Zanna M. P., vol. 23 (San Diego, CA: Academic Press), 1–74.

[ref25] Grigoryev D. Fiske S. T. Batkhina A. (2019). Mapping ethnic stereotypes and their antecedents in Russia: the stereotype content model. Front. Psychol. 10:1643. doi: 10.3389/fpsyg.2019.01643, 31379677 PMC6646730

[ref26] Guassi Moreira J. F. Van Bavel J. J. Telzer E. H. (2017). The neural development of “us and them”. Soc. Cogn. Affect. Neurosci. 12, 184–196. doi: 10.1093/scan/nsw134, 27633395 PMC5488789

[ref27] Hamilton D. L. Trolier T. K. (1986). “Stereotypes and stereotyping: an overview of the cognitive approach,” in Prejudice, Discrimination, and Racism, eds. Dovidio J. F. Gaertner S. L. (Orlando, FL: Academic Press), 127–163.

[ref28] Harris L. T. Fiske S. T. (2006). Dehumanizing the lowest of the low: neuroimaging responses to extreme out-groups. Psychol. Sci. 17, 847–853. doi: 10.1111/j.1467-9280.2006.01793.x, 17100784

[ref29] Haslam N. (2006). Dehumanization: an integrative review. Personal. Soc. Psychol. Rev. 10, 252–264. doi: 10.1207/s15327957pspr1003_4, 16859440

[ref30] Heleven E. Boukhlal S. Van Overwalle F. (2018). A stranger in my brain: neural representation for unfamiliar persons using fMRI repetition suppression. Soc. Neurosci. 13, 530–540. doi: 10.1080/17470919.2017.1358663, 28768460

[ref31] Heleven E. Van Overwalle F. (2015). The person within: memory codes for persons and traits using fMRI repetition suppression. Soc. Cogn. Affect. Neurosci. 11, 159–171. doi: 10.1093/scan/nsv100, 26371337 PMC4692324

[ref32] Heleven E. Van Overwalle F. (2018). The neural basis of representing others’ inner states. Curr. Opin. Psychol. 23, 98–103. doi: 10.1016/j.copsyc.2018.02.003, 29501981

[ref33] Heleven E. Van Overwalle F. (2019a). Neural representations of others in the medial prefrontal cortex do not depend on our knowledge about them. Soc. Neurosci. 14, 286–299. doi: 10.1080/17470919.2018.1472139, 29733764

[ref34] Heleven E. Van Overwalle F. (2019b). The neural representation of the self in relation to close others using fMRI repetition suppression. Soc. Neurosci. 14, 717–728. doi: 10.1080/17470919.2019.1581657, 30757958

[ref35] Henson R. N. (2016). Repetition suppression to faces in the fusiform face area: a personal and dynamic journey. Cortex 80, 174–184. doi: 10.1016/j.cortex.2015.09.012, 26615518

[ref36] Henson R. N. A. Rugg M. D. (2003). Neural response suppression, haemodynamic repetition effects, and behavioural priming. Neuropsychologia 41, 263–270. doi: 10.1016/S0028-3932(02)00159-8, 12457752

[ref37] Holbrook C. Izuma K. Deblieck C. Fessler D. M. T. Iacoboni M. (2016). Neuromodulation of group prejudice and religious belief. Soc. Cogn. Affect. Neurosci. 11, 387–394. doi: 10.1093/scan/nsv107, 26341901 PMC4769621

[ref38] Isik L. Koldewyn K. Beeler D. Kanwisher N. (2017). Perceiving social interactions in the posterior superior temporal sulcus. Proc. Natl. Acad. Sci. USA 114, E9145–E9152. doi: 10.1073/pnas.1714471114, 29073111 PMC5664556

[ref39] Jylhä K. M. Rydgren J. Strimling P. (2022). Xenophobia among radical and mainstream right-wing party voters: Prevalence, correlates and influence on party support. Ethnic and Racial Studies, 45, 261–286. doi: 10.1080/01419870.2022.2061866

[ref40] Kobayashi K. Kable J. W. Hsu M. Jenkins A. C. (2022). Neural representations of others’ traits predict social decisions. Proc. Natl. Acad. Sci. 119:22. doi: 10.1073/pnas.2116944119, 35605117 PMC9295729

[ref41] Kotzur P. F. Schäfer S. J. Wagner U. (2019). Meeting a nice asylum seeker: intergroup contact changes stereotype content perceptions and associated emotional prejudices, and encourages solidarity-based collective action intentions. Br. J. Soc. Psychol. 58, 668–690. doi: 10.1111/bjso.12304, 30512181

[ref42] Kriegeskorte N. Simmons W. K. Bellgowan P. S. Baker C. I. (2009). Circular analysis in systems neuroscience: the dangers of double dipping. Nat. Neurosci. 12, 535–540. doi: 10.1038/nn.2303, 19396166 PMC2841687

[ref43] Krott N. R. Krott E. Zeitner I. (2018). Xenophobic attitudes in German police officers: a longitudinal investigation from professional education to practice. Int. J. Police Sci. Manag. 20, 174–184. doi: 10.1177/1461355718788373

[ref44] La Raja R. Schaffner B. F. (2015). Campaign Finance and Political Polarization: When Purists Prevail. Ann Arbor, MI: University of Michigan Press. doi: 10.3998/ump.13855466.0001.001

[ref45] Larsson J. Smith A. T. (2012). fMRI repetition suppression: neuronal adaptation or stimulus expectation? Cereb. Cortex 22, 567–576. doi: 10.1093/cercor/bhr119, 21690262 PMC3278317

[ref46] Lau T. Cikara M. (2017). FMRI repetition suppression during generalized social categorization. Sci. Rep. 7, 1–10. doi: 10.1038/s41598-017-04115-8, 28655903 PMC5487342

[ref47] Li M. Mai Z. Wang S. Feng T. Van Overwalle F. Ma N. (2021). Warmth is more influential than competence: an fMRI repetition suppression study. Brain Imaging Behav. 15, 266–275. doi: 10.1007/s11682-019-00254-w, 31916071

[ref48] Liberman Z. Woodward A. Kinzler K. (2017). The origins of social categorization. Trends Cogn. Sci. 21, 556–568. doi: 10.1016/j.tics.2017.04.004, 28499741 PMC5605918

[ref49] Ma N. Baetens K. Vandekerckhove M. Kestemont J. Fias W. Van Overwalle F. (2014). Traits are represented in the medial prefrontal cortex: an fMRI adaptation study. Soc. Cogn. Affect. Neurosci. 9, 1185–1192. doi: 10.1093/scan/nst098, 23784074 PMC4127023

[ref50] Ma N. Wang S. Yang Q. Feng T. Van Overwalle F. (2016). The neural representation of competence traits: an fMRI study. Sci. Rep. 6:39609. doi: 10.1038/srep39609, 27995988 PMC5172249

[ref51] Macrae C. N. Bodenhausen G. V. (2000). Social cognition: thinking categorically about others. Annu. Rev. Psychol. 51, 93–120. doi: 10.1146/annurev.psych.51.1.93, 10751966

[ref52] Markowitz D. M. Shoots-Reinhard B. Peters E. Silverstein M. C. Goodwin R. Bjälkebring P. (2021). Dehumanization during the COVID-19 pandemic. Front. Psychol. 12:634543. doi: 10.3389/fpsyg.2021.634543, 33643166 PMC7904886

[ref53] Martherus J. L. Martinez A. G. Piff P. K. Theodoridis A. G. (2021). Party animals? Extreme partisan polarization and dehumanization. Polit. Behav. 43, 517–540. doi: 10.1007/s11109-019-09559-4

[ref54] Menon V. (2011). Large-scale brain networks and psychopathology: a unifying triple network model. Trends Cogn. Sci. 15, 483–506. doi: 10.1016/j.tics.2011.08.003, 21908230

[ref55] Merritt C. C. Maccormack J. K. Stein A. G. Lindquist K. A. Muscatell K. A. (2021). The neural underpinnings of intergroup social cognition: an fMRI meta-analysis. Soc. Cogn. Affect. Neurosci. 16, 903–914. doi: 10.1093/scan/nsab034, 33760100 PMC8421705

[ref56] Miller E. K. Erickson C. A. Desimone R. (1996). Neural mechanisms of visual working memory in prefrontal cortex of the macaque. J. Neurosci. 16, 5154–5167. doi: 10.1523/jneurosci.16-16-05154.1996, 8756444 PMC6579322

[ref57] Molenberghs P. (2013). The neuroscience of in-group bias. Neurosci. Biobehav. Rev. 37, 1530–1536. doi: 10.1016/j.neubiorev.2013.06.002, 23769813

[ref58] Molenberghs P. Johnson H. Henry J. D. Mattingley J. B. (2016). Understanding the minds of others: a neuroimaging meta-analysis. Neurosci. Biobehav. Rev. 65, 276–291. doi: 10.1016/j.neubiorev.2016.03.020, 27073047

[ref59] Nastase S. A. Goldstein A. Hasson U. (2020). Keep it real: rethinking the primacy of experimental control in cognitive neuroscience. NeuroImage 222:117254. doi: 10.1016/j.neuroimage.2020.117254, 32800992 PMC7789034

[ref60] Ostrom T. M. Sedikides C. (1992). Out-group homogeneity effects in natural and minimal groups. Psychol. Bull. 112, 536–552. doi: 10.1037/0033-2909.112.3.536

[ref61] Pietraszewski D. (2022). Toward a computational theory of social groups: a finite set of cognitive primitives for representing any and all social groups in the context of conflict. Behav. Brain Sci. 45:e97. doi: 10.1017/S0140525X21000583, 33902764

[ref62] Quattrone G. A. Jones E. E. (1980). The perception of variability within in-groups and out-groups: implications for the law of small numbers. J. Pers. Soc. Psychol. 38, 141–152. doi: 10.1037/0022-3514.38.1.141

[ref63] Rydgren J. (2004). The logic of xenophobia. Rational. Soc. 16, 123–148. doi: 10.1177/1043463104043712

[ref64] Schurz M. Maliske L. Kanske P. (2020). Cross-network interactions in social cognition: a review of findings on task related brain activation and connectivity. Cortex 130, 142–157. doi: 10.1016/j.cortex.2020.05.006, 32653744

[ref65] Schurz M. Radua J. Aichhorn M. Richlan F. Perner J. (2014). Fractionating theory of mind: a meta-analysis of functional brain imaging studies. Neurosci. Biobehav. Rev. 42, 9–34. doi: 10.1016/j.neubiorev.2014.01.009, 24486722

[ref66] Sellaro R. Derks B. Nitsche M. A. Hommel B. Van Den Wildenberg W. P. M. Van Dam K. . (2015). Reducing prejudice through brain stimulation. Brain Stimul. 8, 891–897. doi: 10.1016/j.brs.2015.04.003, 25991081

[ref67] Shackman A. J. Salomons T. V. Slagter H. A. Fox A. S. Winter J. J. Davidson R. J. (2011). The integration of negative affect, pain and cognitive control in the cingulate cortex. Nat. Rev. Neurosci. 12, 154–167. doi: 10.1038/nrn2994, 21331082 PMC3044650

[ref68] Shkurko A. V. (2013). Is social categorization based on relational ingroup/outgroup opposition? A meta-analysis. Soc. Cogn. Affect. Neurosci. 8, 870–877. doi: 10.1093/scan/nss085, 22847948 PMC3831554

[ref69] Summerfield C. Monti J. M. P. Trittschuh E. H. Mesulam M. Egner T. Inserm U. . (2008). Neural repetition suppression reflects fulfilled perceptual expectations. Nat. Neurosci. 11, 1004–1006. 19160497 10.1038/nn.2163 PMC2747248

[ref70] Tajfel H. Billig M. G. Bundy R. P. (1971). Social categorization and intergroup behaviour. Eur. J. Soc. Psychol. 1, 149–178. doi: 10.1002/ejsp.2420010202

[ref71] Taylor S. E. Fiske S. T. Etcoff N. L. Ruderman A. J. (1978). Categorical and contextual bases of person memory and stereotyping. J. Pers. Soc. Psychol. 36, 778–793. doi: 10.1037/0022-3514.36.7.778

[ref72] Turner J. C. Hogg M. A. Oakes P. J. Reicher S. D. Wetherell M. S. (1987). Rediscovering the Social Group: A Self-Categorization Theory. Oxford, England: Basil Blackwell.

[ref73] Van Overwalle F. (2009). Social cognition and the brain: a meta-analysis. Hum. Brain Mapp. 30, 829–858. doi: 10.1002/hbm.20547, 18381770 PMC6870808

[ref74] Van Overwalle F. Baetens K. (2009). Understanding others’ actions and goals by mirror and mentalizing systems: a meta-analysis. NeuroImage 48, 564–584. doi: 10.1016/j.neuroimage.2009.06.009, 19524046

[ref75] Van Overwalle F. Vandekerckhove M. (2013). Implicit and explicit social mentalizing: dual processes driven by a shared neural network. Front. Hum. Neurosci. 7, 1–6. doi: 10.3389/fnhum.2013.00560, 24062663 PMC3772308

[ref76] Yang Y. White K. R. G. Fan X. Xu Q. Chen Q. (2020). Differences in explicit stereotype activation among social groups based on the stereotype content model: behavioral and electrophysiological evidence in Chinese sample. Brain Sci. 10:1001. doi: 10.3390/brainsci10121001, 33348655 PMC7767265

[ref77] Yee E. Drucker D. M. Thompson-Schill S. L. (2010). fMRI-adaptation evidence of overlapping neural representations for objects related in function or manipulation. NeuroImage 50, 753–763. doi: 10.1016/j.neuroimage.2009.12.036, 20034582 PMC2836190

[ref78] Yeo B. T. T. Krienen F. M. Sepulcre J. Sabuncu M. R. Lashkari D. Hollinshead M. . (2011). The organization of the human cerebral cortex estimated by intrinsic functional connectivity. J. Neurophysiol. 106, 1125–1165. doi: 10.1152/jn.00338.2011, 21653723 PMC3174820

